# Cycling of block copolymer composites with lithium-conducting ceramic nanoparticles

**DOI:** 10.3389/fchem.2023.1199677

**Published:** 2023-06-02

**Authors:** Vivaan Patel, Michael A. Dato, Saheli Chakraborty, Xi Jiang, Min Chen, Matthew Moy, Xiaopeng Yu, Jacqueline A. Maslyn, Linhua Hu, Jordi Cabana, Nitash P. Balsara

**Affiliations:** ^1^ Department of Chemical and Biomolecular Engineering, University of California, Berkeley, Berkeley, CA, United States; ^2^ Materials Sciences Division, Lawrence Berkeley National Laboratory, Berkeley, CA, United States; ^3^ Department of Chemistry, University of Illinois at Chicago, Chicago, IL, United States; ^4^ Department of Materials Science and Engineering, University of California, Berkeley, Berkeley, CA, United States; ^5^ Energy Storage and Distributed Resources Division, Lawrence Berkeley National Laboratory, Berkeley, CA, United States

**Keywords:** composite electrolyte, lithium metal anode, block copolymer electrolyte, ceramic electrolyte, x-ray tomography, LLTO, cell cycling behavior

## Abstract

Solid polymer and perovskite-type ceramic electrolytes have both shown promise in advancing solid-state lithium metal batteries. Despite their favorable interfacial stability against lithium metal, polymer electrolytes face issues due to their low ionic conductivity and poor mechanical strength. Highly conductive and mechanically robust ceramics, on the other hand, cannot physically remain in contact with redox-active particles that expand and contract during charge-discharge cycles unless excessive pressures are used. To overcome the disadvantages of each material, polymer-ceramic composites can be formed; however, depletion interactions will always lead to aggregation of the ceramic particles if a homopolymer above its melting temperature is used. In this study, we incorporate Li_0.33_La_0.56_TiO_3_ (LLTO) nanoparticles into a block copolymer, polystyrene-*b*-poly (ethylene oxide) (SEO), to develop a polymer-composite electrolyte (SEO-LLTO). TEMs of the same nanoparticles in polyethylene oxide (PEO) show highly aggregated particles whereas a significant fraction of the nanoparticles are dispersed within the PEO-rich lamellae of the SEO-LLTO electrolyte. We use synchrotron hard x-ray microtomography to study the cell failure and interfacial stability of SEO-LLTO in cycled lithium-lithium symmetric cells. Three-dimensional tomograms reveal the formation of large globular lithium structures in the vicinity of the LLTO aggregates. Encasing the SEO-LLTO between layers of SEO to form a “sandwich” electrolyte, we prevent direct contact of LLTO with lithium metal, which allows for the passage of seven-fold higher current densities without signatures of lithium deposition around LLTO. We posit that eliminating particle clustering and direct contact of LLTO and lithium metal through dry processing techniques is crucial to enabling composite electrolytes.

## Introduction

The growing need for high-energy density rechargeable batteries has pushed research efforts toward developing solutions to enable the use of lithium metal anodes (Tarascon and Armand, 2001; Trahey et al., 2020). Traditional organic liquid electrolyte systems pose large safety concerns due to electrolyte leakage, thermal stability, and unstable “dendrite” growth when paired with pure lithium metal (Fringant et al., 1995; Agrawal and Pandey, 2008; Cheng et al., 2017). Polymer electrolytes such as poly (ethylene) oxide (PEO) have proven to be a promising alternative due to their ability to solvate lithium ions for beneficial ion transport and their viscoelastic nature leading to good interfacial contact and compatibility with lithium metal electrodes. However, due to the crystalline nature of PEO at room temperature, these electrolytes must be operated well above the melting temperature of PEO resulting in poor mechanical stability (Fenton et al., 1973; Cheng et al., 2014). Various approaches have been taken to improve the mechanical and electrochemical performance of PEO-based electrolytes such as polymer crosslinking and the addition of plasticizers (Fergus, 2010; Qian et al., 2021). Block copolymers offer one avenue to improve the mechanical rigidity of a conducting PEO phase by covalently linking a mechanically rigid block such as polystyrene (PS) to it. While several studies have shown the efficacy of block copolymer electrolytes in hindering the growth of unstable lithium deposition, ion transport is compromised due to the presence of the nonconducting PS block (Devaux et al., 2015; Galluzzo et al., 2020).

Solid perovskite-type ceramic electrolytes are a group of materials that demonstrate high modulus and act as fast ionic conductors (Kato et al., 2016; Zhang et al., 2018). Reported conductivities of polycrystalline lithium lanthanum titanate (LLTO) ranges between 10^−3^–10^−5^ S cm^−1^ at room temperature, where lithium mobility hinges on both the morphology and phase (Inaguma et al., 1993; Stramare et al., 2003; Wang et al., 2004). In spite of high conductivity, the brittleness and lack of compliance of materials like LLTO lead to complications when they are used in batteries with lithium metal anodes. The complications include void formation, poor interfacial adhesion, and electrochemical instability (Chen and Amine, 2001; Wolfenstine et al., 2018; Famprikis et al., 2019). Several researchers have explored the properties of composite electrolytes obtained by dispersing particles of inorganic solid conductors in matrices of ionically conductive polymers (Liu et al., 2015; Li et al., 2019; Kloker et al., 2022). In these systems, both the particles and the polymer matrix are expected to participate in lithium-ion transport. While the addition of LLTO to PEO has been shown to lead an increase in conductivity, the extent to which this increase is due to factors such as reduced crystallization of the polymer remain unresolved (Choi et al., 2015; Chen et al., 2018). Milian Pila et al. (2019) showed that the conductivity of PEO-LLTO composites increases with LLTO addition, but only up to 10 weight percent LLTO. They found a decrease in conductivity when the LLTO weight percent was increased to 15%. It is common to gain insight into lithium transference in electrolytes by conducting constant potential experiments in a lithium-electrolyte-lithium symmetric cell and measuring the initial current, *i*_0_, and the steady-state current, *i*_ss_. We define the current fraction, *ρ*_+_ = *i*_ss_/*i*_0_ (Bruce and Vincent, 1987; Bruce et al., 1988; 1992). Liu et al. (2019) report higher values of *ρ*_+_ in PEO-LLTO composites relative to PEO. Symmetric cells with PEO-LLTO composite electrolytes exhibit higher cycling stability when compared with PEO electrolytes.

While the morphology of the ceramic particles prior to their addition into the polymer is generally known, the extent to which they are aggregated is difficult to predict and quantify. Aggregation in polymer-ceramic composites is driven by depletion interactions. When the distance between two adjacent particles is less than the radius of gyration of the polymer chains, the chains are propelled into the matrix by entropic driving forces, and irreversible aggregation of the particles (Gast et al., 1983). Thus, processing steps will dictate the morphology of the polymer-ceramic composite, especially if a solution-casting process that requires evaporating solvent is used to form the composite. In addition, it is unclear if the passage of ionic current results in morphological changes within the electrolyte, or at the electrode/electrolyte interface.

In this work, we study the electrochemical performance of a block copolymer (PS-*b*-PEO or SEO) composite with cubic phase LLTO nanoparticles. We posit that the use of a block copolymer reduces depletion interactions; the mechanism for this reduction will be discussed later. We present measurements of conductivity and current fraction of composite electrolytes. The state of aggregation of the nanoparticles is analyzed using transmission electron microscopy and hard x-ray microtomography. Our main objective is to evaluate the ability of these composites to sustain dc current in lithium-electrolyte-lithium symmetric cells. Morphological changes within the composite electrolyte and at the electrode-electrolyte interfaces were investigated using hard x-ray microtomography. The morphological changes provide a mechanistic understanding of the factors that limit the ability of the composites to sustain dc current.

## Materials and methods

### Synthesis

#### Lithium lanthanum titanate (LLTO) synthesis

Chemicals: ethanol (200 proof); Ethylene glycol (99%); lithium acetate (99%); lanthanum (III) nitrate hexahydrate (99.999%); oleic acid (90%); sodium hydroxide (97%); titanium (IV) butoxide (97%). All chemicals were purchased from Sigma-Aldrich and used as received.

In a typical synthesis, 0.75 mmol of lithium and lanthanum precursors were dissolved in 15 mL of DI water, making Solution 1. Then, 1.2 g of sodium hydroxide was dissolved in 6 mL of DI water, making Solution 2. To make Solution 3, 1.5 mmol of titanium (IV) butoxide was dissolved in 52.5 mL of absolute ethanol in an argon-filled glovebox. The solution was removed, then 7.5 mL of oleic acid was added and stirred under ambient conditions. To create the reaction slurry, Solutions 1 and 2 were added sequentially to Solution 3 under stirring in open air. The addition of Solution 1 induced a white precipitation due to a rapid change in solubility of the salts, and the addition of Solution 2 resulted in a total reaction mixture at pH 13. The resulting slurry was transferred to a 125 mL Teflon-lined autoclave (Parr Instrument Co., No. 4748). This was sealed and placed in an oven ramped to 240°C at a rate of 4°C/min, where the time at-temperature was 18 h. The vessel was allowed to cool ambiently to room temperature. Then, the reaction mixture was sequestered, and product washed with ethanol five times, collected by centrifugation. The resulting white powder was allowed to dry in an oven at 80°C overnight. Phase identification was performed via Powder X-Ray Diffraction (PXRD) with a Bruker D8 Advance under Bragg-Brentano geometry, Cu K-α radiation (K-α_1_ = 1.54059Å, K-α_2_ = 1.54443Å) at 40 kV and 40 mA and a slit width of 0.681 mm. Coupled-2θ angles varied from 10° to 90° at 0.01°/step with exposure time of 0.850 s. To confirm phase identity and purity, Pawley Refinements was executed using the Jana2006 software package. Tracking size and shape of the nanocrystalline domains before implementation into the hybrid was performed via Transmission Electron Microscopy (TEM) measurements using a JEOL-3,010 microscope operating at 300 kV on 400-mesh lacey carbon copper-backed grids (Ted Pella Inc.) in the bright field mode. Using a helium gas pycnometer, the density of the resulting powder was calculated to be 4.71 g cm^−3^.

#### Block copolymer (SEO) synthesis

In this study, the polystyrene-*b*-poly (ethylene oxide) (SEO) polymers were synthesized following the method of anionic polymerization described in previous work (Maslyn et al., 2018). The molecular weight of the polystyrene (PS) block and the poly (ethylene oxide) (PEO) block is 200 and 222 kg mol^−1^, respectively.

### Electrolyte preparation

To remove any residual moisture, the PEO homopolymer, LLTO, and LiTFSI were dried under active vacuum at 90°C for 48 h in the glovebox antechamber before transferring into the glovebox. The SEO copolymer was similarly dried at 120°C. All sample preparation was conducted in an argon glovebox (MBraun) where the H_2_O and O_2_ levels were both maintained to be less than 0.1 ppm.

The SEO dry polymer and LiTFSI salt were dissolved in n-methyl pyrollidine (NMP) and stirred at 100°C until fully dissolved. The solution was free-cast onto a heated casting plate lined with nickel foil under vacuum at 60°C. The resulting transparent film was dried under vacuum at 120°C for 48 h. The salt concentration, *r*, was defined as the ratio between lithium and ethylene oxide monomer units (*r* = [Li]/[EO]), where the salt is assumed to only reside in the EO domains. The electrolytes used in this study have a salt concentration of *r* = 0.085.

SEO-LLTO composite electrolytes were prepared by dissolving the dry polymer and LiTFSI salt in NMP at 120°C. The salt concentration was fixed to *r* = 0.085 relative to the SEO polymer. The LLTO powder was subsequently added and mixed using a homogenizer at room temperature to reduce the amount of aggregation during casting. The resulting cloudy solution was similarly free-cast on a heating casting plate (60°C) and the final film was dried under vacuum at 120°C for 48 h. It is important to characterize the change in the conducting phase volume fraction of the composite electrolyte. Given that the LLTO particles are ionically conductive, we can calculate the conducting phase volume fraction, $\phi_{c}$ , using the following equation:

$$\phi_{c}=\frac{\nu_{EO}+\gamma \nu_{LLTO}+r\nu_{LiTFSI}}{\nu_{EO}+\gamma \nu_{LLTO}+r\nu_{LiTFSI}+\frac{M_{PS}M_{EO}}{M_{S}M_{PEO}}\nu_{S}}$$ (1)

where *ν*_EO_, *ν*_S_, *ν*_LLTO_, and *ν*_LiTFSI_ is the molar volume of ethylene oxide, styrene, LLTO, and LiTFSI, respectively. *M*_PS_, *M*_EO_, *M*_S_, *M*_PEO_ are the molecular weights of polystyrene, ethylene oxide, styrene, and polyethylene oxide, respectively. The specific values were taken from previous work (Maslyn et al., 2021). Since we assume the LLTO resides predominantly in the PEO domains, we define the LLTO concentration, *γ*, as the ratio between LLTO and ethylene oxide monomer units (*γ* = [LLTO]/[EO]). The salt concentration was fixed to *r* = 0.085. Table 1 outlines the series of composite electrolytes with varying weight fractions of LLTO, *w*_LLTO_, used in this study. Despite the addition of 34 weight percent of LLTO, there is only a nominal increase in volume fraction owing to the high density of LLTO particles compared to SEO. By assuming the LLTO only resides in the PEO domain, we can similarly calculate the volume fraction of only the LLTO present within the PEO-rich microphase denoted as $\phi_{LLTO}$ .

$$\phi_{LLTO}=\frac{\gamma \nu_{LLTO}}{\nu_{EO}+\gamma \nu_{LLTO}+r\nu_{LiTFSI}}$$ (2)

**TABLE 1:** Volume fraction of SEO-LLTO composite electrolytes at a fixed salt concentration.

Composite	*w*_LLTO_	*w*_LLTO,PEO_	$\phi_{c}$	$\phi_{LLTO}$
SEO-LLTO-34	0.34	0.50	0.61	0.15
SEO-LLTO-26	0.26	0.40	0.60	0.10
SEO-LLTO-18	0.18	0.30	0.59	0.07
SEO-LLTO-12	0.12	0.20	0.58	0.04
SEO-LLTO-5	0.05	0.10	0.575	0.02
SEO	0	0	0.57	0

At the highest LLTO weight percent, the LLTO particles only comprise 15% of the entire PEO domain. By assuming the LLTO is predominantly in the PEO domain, we can approximate the weight fraction of LLTO with respect to the volume fraction of PEO in the block copolymer as *w*_LLTO,PEO_.

$$w_{LLTO,PEO}=\frac{m_{LLTO}}{m_{SEO}\phi_{EO}+m_{LLTO}}$$ (3)

We assume the density of EO and SEO to be comparable in this calculation. We can calculate the volume fraction of ethylene oxide, $\phi_{EO}$ , in a similar fashion as $\phi_{c}$ but ignoring the LiTFSI and LLTO components.

A neat PEO-LLTO composite was formed by dissolving PEO with a molecular weight of 35 kg mol^−1^ in NMP and stirred at 100°C. Subsequently, LLTO particles were added and mixed using a homogenizer at room temperature. The resulting mixture was heated at 100°C overnight followed by a drying step at 120°C under vacuum for 48 h to remove residual NMP.

A sandwich electrolyte was made by layering a 5/16 in. SEO electrolyte onto a SEO-LLTO electrolyte disc in a heated hand press at 120°C. Once the electrolyte had merged together, another SEO electrolyte was layered on the other side and placed back in the hand press. The entire stack was heated at 120°C for 3 min before using in cell assembly.

### Differential scanning calorimetry

Polymer electrolytes were hermetically sealed in a Tzero aluminum pan inside an argon glovebox. Differential scanning calorimetry (DSC) thermograms were obtained using the Thermal Advantage Q200 calorimeter at the Molecular Foundry, Lawrence Berkeley National Laboratory. The salty electrolytes were heated to 150°C at 10°C/min then cooled to −75°C at 5°C/min followed by a final heating cycle to 150°C at 10°C/min. Baseline corrections were applied to the heating curves. Melting temperatures and enthalpies were obtained from the second heating cycle using the TA universal Analysis software. The percent crystallinity, χ_PEO_, was calculated using

$$\chi_{PEO}=\frac{\Delta H_{m}}{\Delta H_{m,PEO}},$$ (4)

where Δ*H*_m_ is the enthalpy of melting of the sample, and Δ*H*_m,PEO_ is the enthalpy of melting of a pure crystalline PEO sample. For this analysis, we use Δ*H*_m,PEO_ = 214 J g^−1^ (Beaumont et al., 1966)

### Rheology

A stress-controlled Anton-Paar MCR 302 Rheometer with 8 mm diameter parallel plates was used to measure the viscoelastic properties of the polymer samples. The samples were prepared by hot-pressing neat polymers at 120°C for 24 h in a rubber space that has a thickness of 1 mm and an internal diameter of 8 mm. To equilibrate the grain structure in the rheometer, the samples were further annealed between the plates of the rheometer at 120°C overnight. A frequency sweep experiment between 0.1 rad/s and 100 rad/s was conducted at 90°C to obtain the storage (*G′*) and loss (*G″*) modulus for each sample. The strain rate was fixed at 0.01%.

### Electrochemical techniques

#### Ionic conductivity

Aluminum blocking electrode cells were assembled inside an argon glovebox and used to obtain ionic conductivities of the electrolytes using AC impedance spectroscopy. The casted film was to punch out 5/16 in. diameter electrolyte discs. The thickness of the electrolyte was measured using a micrometer. Similarly, aluminum foil was used to obtain electrodes with a diameter of 1/4 in. Two aluminum electrodes were mechanically pressed on either side of the electrolyte. Aluminum tabs were used as current collectors and placed over the blocking electrodes. The entire cell was vacuum sealed in the pouch material.

A Biologic VMP3 potentiostat was used to obtain complex AC impedance spectroscopy measurements for a frequency range of 1MHz–100 mHz with an amplitude of 50 mV. The Nyquist plot data were fit to an equivalent circuit to extract out the value for the bulk impedance, *R*_b_. The ionic conductivity, *κ*, was calculated using

$$\kappa =\frac{L_{el}}{aR_{b}},$$ (5)

where *a* is the area of the blocking electrode and *L*_*el*_ is the thickness of the electrolyte measured using a micrometer. All measurements were taken at 90°C.

#### Current fraction

Lithium-electrolyte-lithium symmetric cells were assembled inside an argon glovebox and used to obtain current fraction measurements. For current fraction experiments, two 1/4 in. Lithium electrodes were punched out from lithium metal foil. 5/16 in. diameter electrolyte discs were punched out from the casted film, sandwiched between the lithium electrodes, and mechanically pressed. Nickel tabs were placed over the lithium metal and the entire assembly was vacuum sealed in the pouch material.

Before conducting any experiments, the cells were annealed at 120°C for 4 h. Cells were then pre-conditioned using eight charge/discharge cycles with a current density of 0.02 mA cm^−2^. Each cycle had a 4 h charging period, 45 min rest, 4 h discharging period, and a 45 min rest. The steady state current experiments were carried out by polarizing the cells at a constant potential, Δ*Φ*, for 4 h until a steady-state current, *i*_SS_, was reached. The bulk and interfacial impedance (*R*_*b*_ and *R*_*i*_) was measured every hour using ac impedance spectroscopy. To ensure the measurements did not depend on the magnitude and sign of the applied voltage, −10 mV, 10 mV, −20 mV, and 20 mV were applied consecutively. All the measurements were taken at 90°C. The initial current, *i*_Ω_, is calculated using Ohm’s Law in Eq. 6:

$$i_{\Omega }=\frac{\Delta \Phi }{R_{i,0}+R_{b,0}},$$ (6)

*R*_b,0_ and *R*_i,0_ represent the initial bulk and interfacial resistances, respectively. The Bruce-Vincent (Bruce and Vincent, 1987; Bruce et al., 1988; 1992) method was used to calculate the steady state current fraction, *ρ*_*+*_, using the following equation:

$$\rho_{+}=\frac{i_{ss}\left(∆\Phi -i_{\Omega }R_{i,0}\right)}{i_{\Omega }\left(∆\Phi -i_{ss}R_{i,ss}\right)}\;.$$ (7)

#### Constant-current polarization

To assemble cells that were appropriate for imaging using x-ray microtomography, a stack of three sheets of lithium foil and one sheet nickel foil were used as the electrodes. The nickel foil ensures even current distribution and provides better mechanical support. 5/16 in. diameter electrolytes were punched from the casted film and placed between two 1/4 in lithium electrodes. The entire cell was placed between two stainless steel shims attached to aluminum tabs as current collectors. The cell was vacuum sealed in the pouch material. The pouch cells were annealed and pre-conditioned using the protocol described previously. Cells were then polarized at varying current densities in alternating directions until cell failure. Polarization time was minimized to reduce the effect of lithium dendrite growth in our cells. Electrochemical impedance spectroscopy was taken before and after each polarization step to ensure stable bulk and interfacial impedances. All experiments were conducted at 90°C.

### Synchrotron X-ray microtomography

The cells were imaged using hard X-ray microtomography at the Advanced Light Source (ALS) beamline 8.3.2. Monochromatic x-rays with an energy of 22 keV were transmitted through the sample and converted to visible light using a scintillator. The image was magnified using a ×2 or ×4 lens and converted to a digital image using an optical microscope. The pixel size is approximately 3.25 and 1.62 μm for a ×2 and ×4 lens, respectively. The sample was incrementally rotated 180° to collect a total of 1,313 projections. Using similar protocols outlined in previous work, cross-sectional slices were reconstructed and then rendered using ImageJ. Three-dimensional displays (3D) were constructed using the Avizo software package.

### Transmission electron microscopy

The electrolytes were sectioned at −120°C using a cryomicrotome (Leica Ultracut 6) to obtain ultrathin films (∼100 nm). The films were then transferred onto copper grids coated with lacey carbon support films. After warming up under the protection of nitrogen atmosphere in the cryomicrotome chamber, the grids were transferred immediately inside the glovebox to prevent absorption of moisture. The sections of electrolytes were stained by ruthenium tetroxide (RuO_4_) vapor for 5 min to improve the contrast (PEO block are shown in bright in micrographs). High-angle annular dark-field scanning transmission electron microscopy (HAADF-STEM) micrographs and elemental maps were collected using the FEI TitanX 300 kV with a camera length of 190 cm and energy-dispersive X-ray spectroscopy (EDS), respectively.

## Results and discussion

**FIGURE 1:**
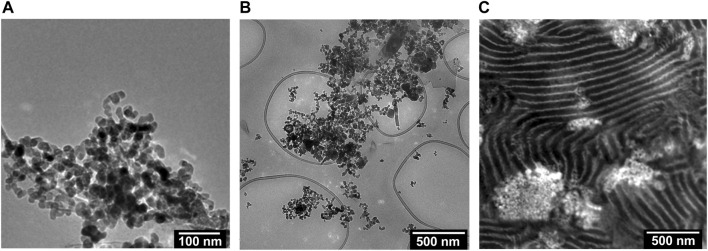
TEM images of the **(A)** LLTO particles dispersed in ethanol, **(B)** a composite of PEO and LLTO (w_LLTO_ = 0.4), and **(C)** a RuO_4_ stained composite of SEO and LLTO (w_LLTO_ = 0.26). The LLTO particles have an average diameter of around 30 nm. In **(C)**, the bright regions correspond to PEO domains and dark regions represent PS domains forming a lamellar morphology. The LLTO nanoparticles appear brighter than PEO and are located mostly in the PEO lamellae.

**FIGURE 2:**
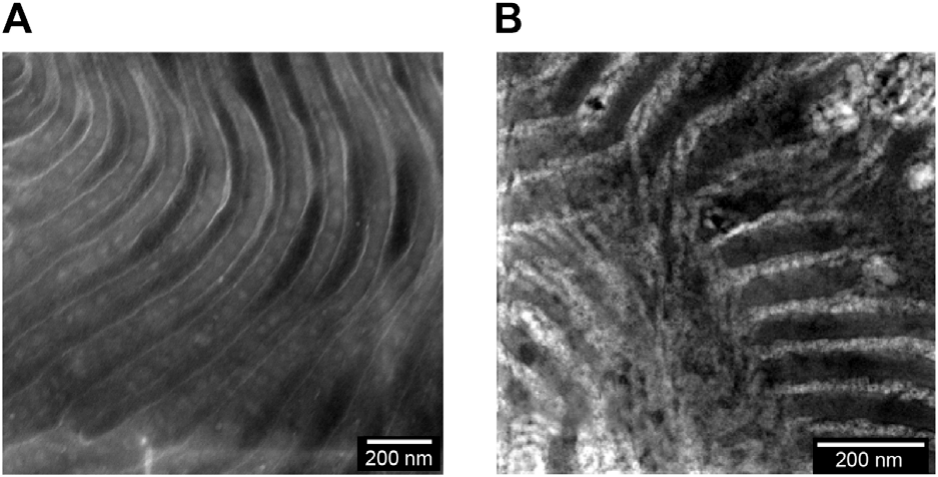
RuO_4_ stained TEM images of the membrane surfaces are shown for the **(A)** neat SEO and **(B)** a composite of SEO and LLTO (w_LLTO_ = 0.26). In **(A)** and **(B)**, the bright regions correspond to PEO domains and dark regions represent PS domains forming a lamellar morphology. The LLTO nanoparticles appear brighter than PEO and are located mostly in the PEO lamellae.

We begin by describing the morphology of our composite electrolytes based on TEM. Due to the hygroscopic nature of LiTFSI salt, all the TEM images were obtained without salt. Figure 1A shows a TEM image of the LLTO nanoparticles deposited on a carbon substrate from an ethanol solution. We see spherical nanoparticles with an average diameter of about 30 nm and a narrow size distribution. The aggregation of these particles probably occurred during the centrifugation and washing steps. Figure 1B shows a TEM image of a composite containing PEO and LLTO. The PEO matrix is transparent in this TEM while the LLTO particles are clustered within a large aggregate. It is evident that our solution processing and drying steps results in extensive aggregation of the LLTO particles in PEO. There are very few unaggregated nanoparticles in the PEO-LLTO sample. Figure 1C shows a TEM image of a typical SEO composite containing LLTO. The weight fraction of LLTO with respect to the PEO volume fraction is comparable to the homopolymer composite. Here we see alternating bright (PEO-rich) and dark (PS-rich) lamellar domains with a periodicity of around 110 nm. We also see bright clusters of LLTO, but these clusters are dispersed in the polymer. A small nanoparticle size distribution achieved with our sol-gel method is crucial for dispersibility within these PEO lamellae. Figures 2A, B show higher resolution TEM images of the neat SEO and the composite shown in Figure 1C. The lamellar morphology is preserved upon the addition of LLTO. Dense collections of LLTO particles are seen within the PEO lamellae. Since their electron density is higher than PEO, they appear brighter than PEO in the TEM image. The fact that most of the LLTO particles lie within the PEO lamellae suggests favorable thermodynamic interactions between LLTO and PEO relative to those between LLTO and PS. Further work is needed to determine the molecular underpinnings of these interactions. The images in Figures 1, 2 show that aggregation of LLTO in polymer films due to depletion interactions is suppressed by using the SEO block copolymer instead of the PEO homopolymer.

**FIGURE 3:**
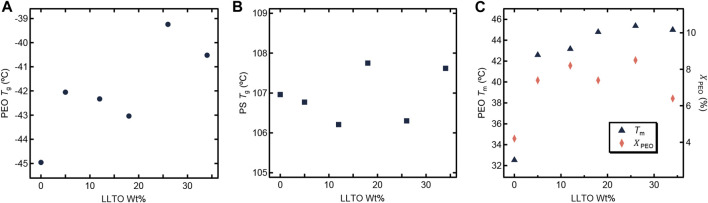
Thermal characteristics of the SEO-LLTO composite samples at *r* = 0.085 is plotted as a function of LLTO weight percentage. **(A)** The glass transition temperature (*T_g_*) of PEO is represented as a dark filled circle. **(B)** The *T_g_* of PS is shown in dark filled squares. **(C)** The melting temperature (*T_m_*) of PEO is shown in dark filled triangles and the crystallinity of the PEO domain (*χ_PEO_*) is represented as filled orange diamonds.

We now turn to the physical and electrochemical properties of the composite electrolytes listed in Table 1. The composite samples are labelled as SEO-LLTO-x, where x refers to the weight fraction of LLTO. All electrolytes studied contain LiTFSI at *r* = 0.085. Our calculation of *r* is based on the assumption that all of the salt resides in the PEO-rich microphase. Figure 3 shows the thermal characteristics of the SEO-LLTO electrolytes. Upon a small increase in LLTO weight percent, we observe a rise of 3°C in the glass transition temperature of the PEO-rich microphase. After adding close to 30 weight percent LLTO to SEO, however, the glass transition temperature shows a noticeable increase by another 4°C (Figure 3A). The melting temperature of this microphase increases abruptly from 33°C to 43°C even with a nominal increase in weight percent of LLTO from 0% to 5%. Further increase in the weight percent of LLTO leads to a plateau and has a negligible effect on the melting temperature. The crystallinity of the PEO-rich microphase is low (below 10%), presumably due to the presence of LiTFSI. A slight increase in the crystallinity is observed upon addition of LLTO (Figure 3C). The effect of added LLTO on the amorphous PS-rich microphase is negligible as shown in Figure 3B. The data in Figure 3 are consistent with the TEM images (Figure 1C; 2B); they indicate that the particles reside primarily within the PEO-rich domains of the composite.

**FIGURE 4:**
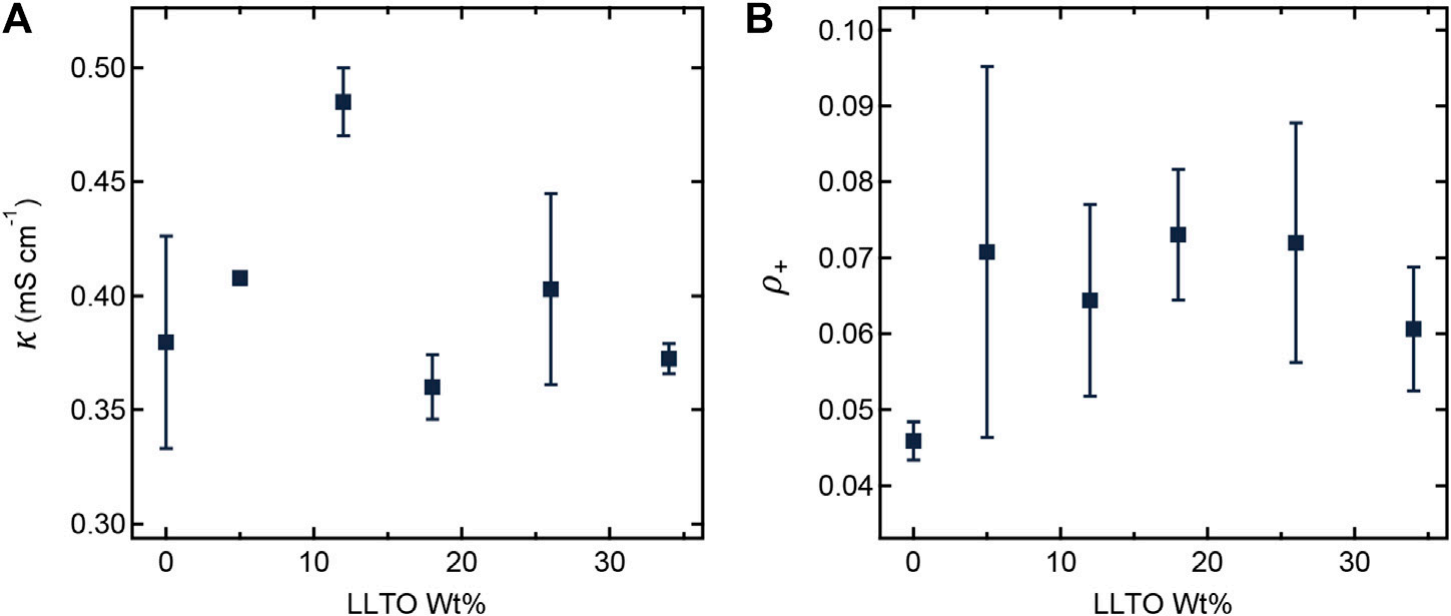
**(A)** Ionic conductivity (*κ*) and **(B)** current fraction (*ρ_+_*) plotted as a function of LLTO weight percentage at 90°C.

Figure 4 shows the dependence of ionic conductivity (*κ*) and current fraction (*ρ*_+_) on LLTO weight percent at 90°C. Since both *κ* and *ρ*_+_ of pure LLTO particles are higher than those of PEO/LiTFSI, we expect increases in *κ* and *ρ*_+_ with increasing LLTO weight percent (Inaguma et al., 1993; Gao and Balsara, 2021). However, such behavior assumes that the interfacial impedance between LLTO and PEO/LiTFSI is negligible. The work of Gupta and Sakamoto, (2019); Kim et al. (2022) shows the importance of this impedance. Based on the data in Figure 4, we conclude this increase is within experimental error for our composites. In other words, both *κ* and *ρ*_+_ are independent of the LLTO weight percent. While the TEM data in Figures 1C, 2B show clearly that the LLTO nanoparticles have been incorporated into our composites, electrochemical characterization data in Figure 4 shows that this did not lead to improvement of ion transport. We posit that the lack of improvement may be a result of small LLTO volume fractions in the conducting domains (see Table 1) and the presence of interfacial impedances that have not yet been characterized. One expects high conductivity in composites where the LLTO particles form a percolating network (Liu et al., 2018; Zheng and Hu, 2018). The sequestration of the LLTO nanoparticles in the PEO domain may help with the formation of percolating nanoparticles and the TEM image in Figure 2B might even suggest that the particles are in close proximity to each other. Yet, the electrochemical data in Figure 4 shows no evidence of percolating nanoparticles.

**FIGURE 5:**
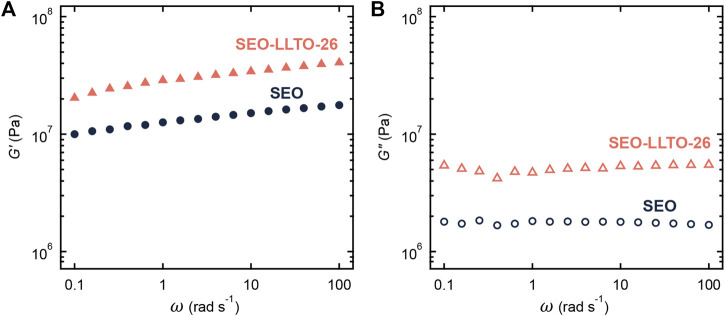
**(A)** Storage modulus (*G′*) and **(B)** loss modulus (*G″*) plotted as a function of frequency for neat SEO (circle) and SEO-LLTO-26 (triangle). All measurements were taken at 90°C.

Figures 5A, B shows the frequency dependence of storage modulus (*G′*) and loss modulus (*G″*) of the neat SEO and SEO-LLTO-26 samples at 90°C (without salt). The weak dependence of *G′* on frequency for both samples indicates solid-like behavior. The *G′* and *G″* of the composite are higher than the SEO. We attribute this to the presence of solid LLTO particles with moduli that orders of magnitude than that of SEO (Stone et al., 2011; Wolfenstine et al., 2018).

**FIGURE 6:**
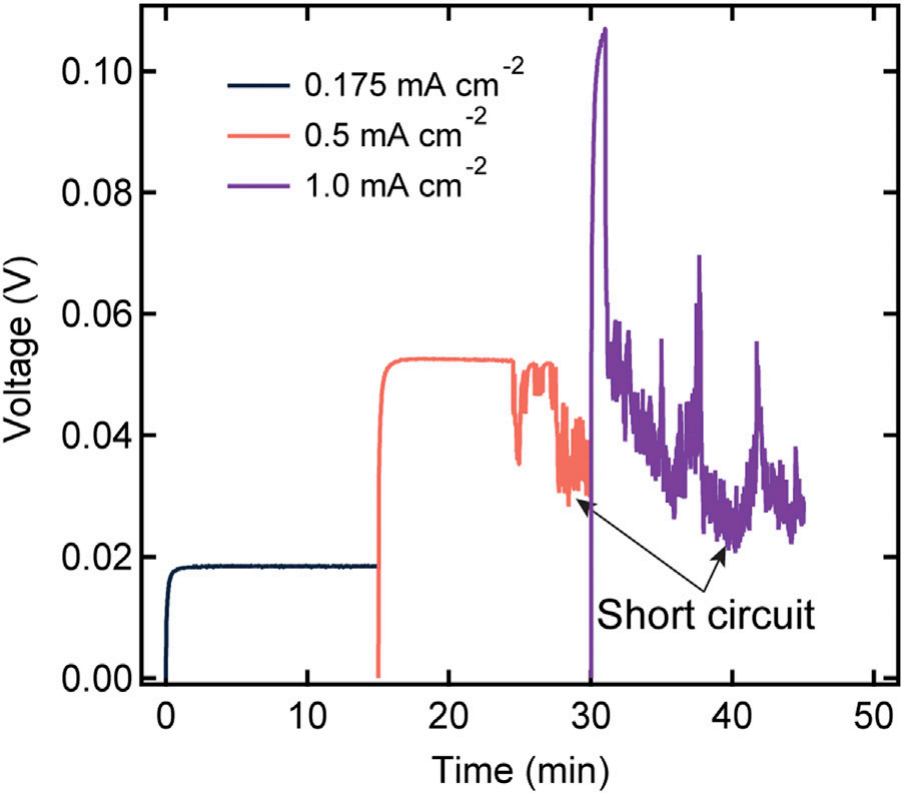
Voltage profiles as a function of time for a SEO-LLTO-26 electrolyte in a lithium symmetric cell at various current densities ranging from 0.175 mA cm^−2^ to 1 mA cm^−2^. The thickness of the polymer-composite electrolyte was 20 μm. The measurement was taken at 90°C.

We now discuss the cycling characteristics of the SEO-LLTO electrolytes. Figure 6 shows the voltage profile of an SEO-LLTO-26 electrolyte at three different current densities. To avoid excessive plating on one electrode during constant current experiments, the direction of polarization was alternated between positive and negative current densities. For simplicity, we chose to show the magnitude of the voltage response in Figure 6. The voltage approached a stable plateau of 0.02 V when a small current density of 0.175 mA cm^−2^ was applied. Subsequently, the magnitude of the current density was increased to 0.5 mA cm^−2^. The voltage initially stabilized at a value of 0.05 V, consistent with the increase in the current density. However, signatures of cell failure were evident after about 10 min of polarization. A further increase in current density to 1 mA cm^−2^ resulted in highly unstable behavior typical of short-circuited cell. It is clear that the SEO-LLTO composite is unable to sustain current densities in the vicinity of 0.5 mA cm^−2^ and higher. In previous studies, we have shown that SEO electrolytes can sustain significantly higher current densities of around 3 mA cm^−2^ (Maslyn et al., 2021; Frenck et al., 2022).

**FIGURE 7:**
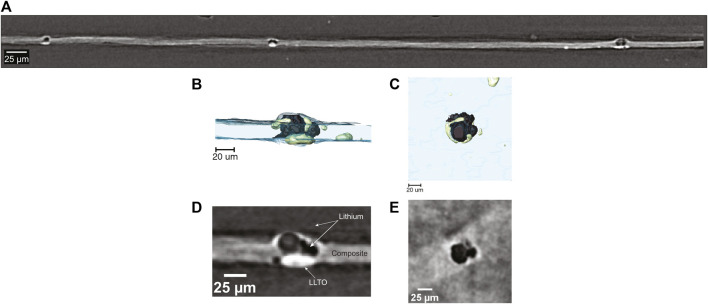
**(A)** Field-of-view tomogram showing formation of lithium globules surrounding areas of high LLTO concentration throughout the SEO-LLTO-26 electrolyte. **(B)** Cross-sectional 3D rendering of lithium deposition (black) around LLTO clusters (green) present within the composite electrolyte (blue). **(C)** Top-down 3D rendering of lithium deposition in the vicinity of LLTO clusters **(D)** A raw cross-sectional tomogram of lithium deposition (black) within the composite (gray) corresponding to the rendering in **(B)**. The LLTO clusters appear white. **(E)** A raw top-down tomogram slice of lithium globule formation depicted in **(C)**.

Figure 7A shows a cross section of the cell in Figure 6 with a wide field-of-view obtained by hard x-ray microtomography. The lithium metal is the component in the cell with the lowest electron density and it appears dark in the cross-section. The composite electrolyte is seen between the two lithium electrodes, and it appears as a light gray band. Since the LLTO particles comprise heavy elements, they appear bright in the tomograms. The bright white spots present within the electrolyte represent clusters of LLTO particles that were not incorporated into the PEO-rich lamellae of the SEO copolymer. These clusters are also evident in the TEM shown in Figure 1C. Large globules that appear black are shown to form directly on top of the bright LLTO clusters. These globules penetrate through the electrolyte and are the cause of cell failure. The number of electrolyte-spanning lithium protrusions is large; three can be seen in Figure 7A, which is a very small portion of the cell. More details on the morphology of the protrusions can be seen in the 3D rendering of the cell in the vicinity of one of the protrusions (Figures 7B, C). The lithium metal, rendered black, is partially enveloped by LLTO clusters depicted in green. The side view presented in Figure 7B confirms that the lithium protrusion spans the electrolyte. While a large LLTO cluster is evident at the base of the protrusion, clusters are also found around the periphery of the protrusion. This is seen more clearly in the rendering of the top-down view (Figure 7C). The two-dimensional slices through the tomogram shown in Figures 7D, E show the raw images that are the basis of the segmentation used to obtain the 3D rendering. These images support our conclusions regarding the morphology of the protrusion.

It has been shown that LLTO is unstable against lithium metal causing the reduction of Ti^4+^ ions to Ti^3+^, which makes the LLTO particles electronically conductive (Bohnke et al., 1996; Birke et al., 1997). We hypothesize that in our SEO-LLTO composites, this happens when current densities of 0.5 mA cm^−2^ are applied. At this current density, lithium ions are reduced on top of these electronically conducting particles. It is probable that some block copolymer material is trapped within an agglomeration of particles. We see many small LLTO clusters in the area surrounding of the lithium protrusion (Figures 7B–E). Thus, it appears as though lithium metal was nucleated within a cluster and the growth of the protrusion resulted in fragmentation of the LLTO cluster. It is clear that the polymer-ceramic composite electrolytes can only be stable against lithium metal if the ceramic clusters are eliminated, especially those in contact with the lithium metal electrode.

**FIGURE 8:**
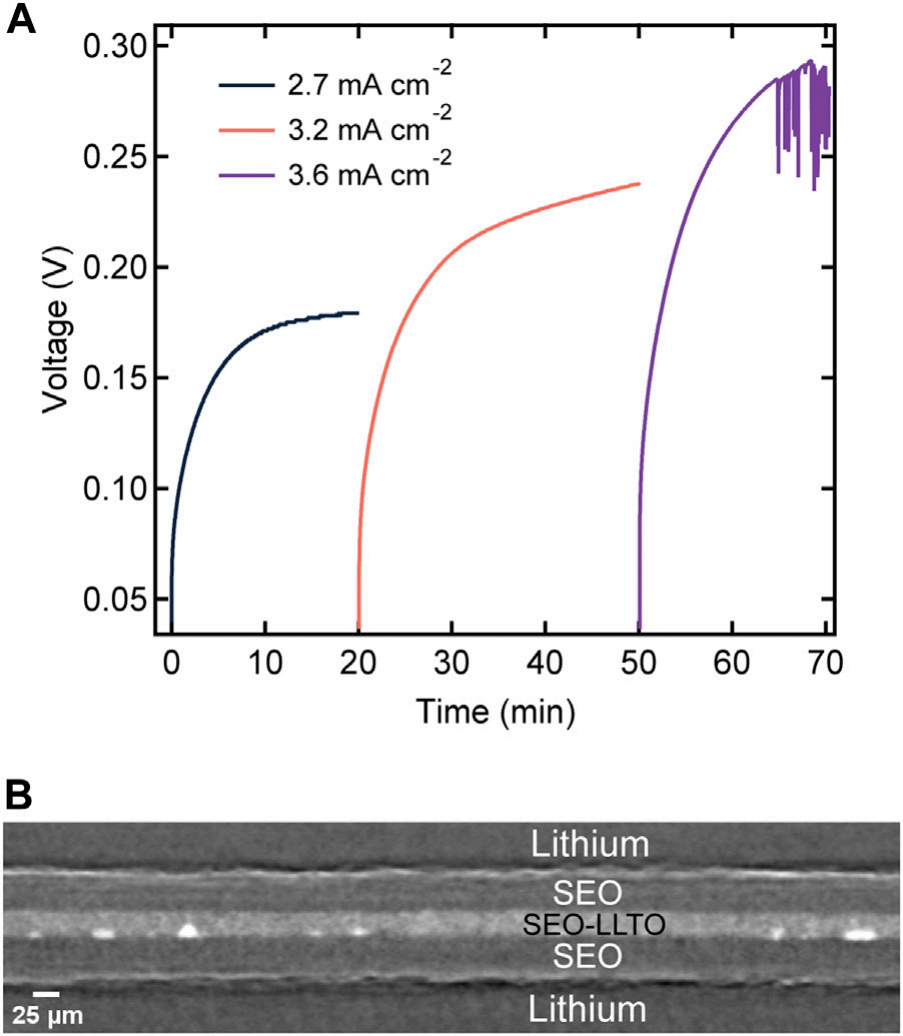
**(A)** Voltage profiles as a function of time for a layered electrolyte comprised of SEO | SEO-LLTO-26 | SEO in a lithium symmetric cell at various normalized current densities ranging from 2.7 mA cm^−2^–3.6 mA cm^−2^ at 90°C. The thickness of the sandwich electrolyte was 90 μm. **(B)** Cross-sectional tomogram of the sandwich electrolyte indicating no globule formation surrounding LLTO particles at high current densities.

To test our hypothesis, we constructed a sandwich electrolyte structure, where we placed the composite electrolyte between two SEO electrolyte films. Both lithium electrodes are thus covered by the SEO electrolyte, and this eliminates contact between the LLTO clusters and lithium metal anode. Figure 8A displays the magnitude of voltage versus time profile for the sandwich electrolyte upon constant current polarizations. To account for differences in electrolyte thicknesses, we calculate a normalized current density, which corresponds to a current density applied to a 20 μm thick electrolyte, using the following equation:

$$i_{norm}=i\left(\frac{L}{20}\right),$$ (8)

where *L* is the total sandwiched electrolyte thickness. This enables direct comparison with the data in Figure 6. We observe stable voltage versus time curves at values of *i*_norm_ as high as 2.7 and 3.2 mA cm^−2^ through the sandwich structure. Evidence for cell failure was only seen at *i*_norm_ = 3.6 mA cm^−2^. This corresponds to a seven-fold increase in current required to reach cell failure.

**FIGURE 9:**
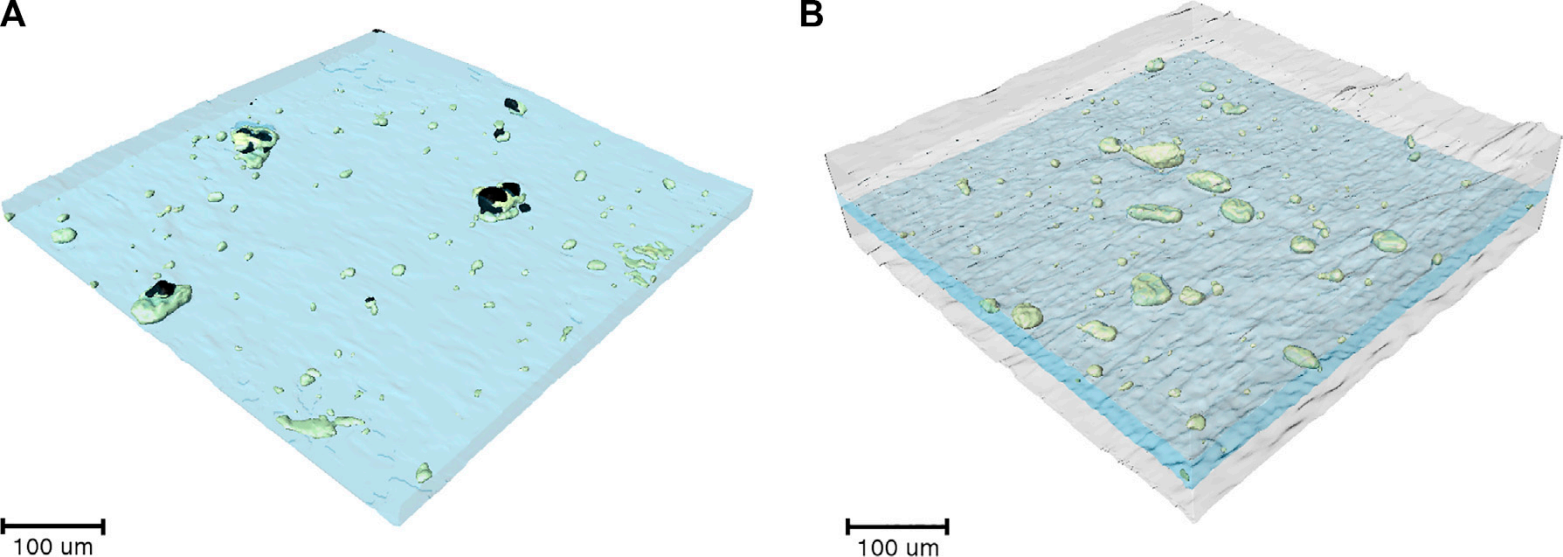
**(A)** 3D rendering of SEO-LLTO-26 electrolyte after cycling. Electrolyte-spanning lithium globules (black) are seen in the vicinity of LLTO clusters (green). **(B)** 3D rendering of the SEO | SEO-LLTO-26 | SEO composite where the LLTO clusters (green) within the composite electrolyte (blue) are sandwiched between SEO layers (gray). No evidence of lithium globules was found.

After the experiments in Figure 8A were completed, the cell was imaged using hard x-ray microtomography. Figure 8B shows a cross-sectional tomogram of the failed sandwich electrolyte. The composite electrolyte in the middle appears brighter than the surrounding SEO electrolyte due to the dispersion of heavy LLTO particles. However, some of the particles were not dispersed and thus, clusters are evident in all our composites including the one shown in Figure 8B. Figures 9A, B show a 3D rendering of a representative area in the SEO-LLTO composite and the sandwich electrolyte after cycling. The difference between Figures 9A, B is stark. We see many system-spanning globules in the composite electrolyte in Figure 9A when the imposed current was only 0.5 mA cm^−2^. In contrast we were unable to find any system-spanning globules in the composite electrolyte in Figure 9B when the imposed current was as high as 3.6 mA cm^−2^. We attribute this to the lack of direct contact between the LLTO particles and lithium metal.

To ensure that the differences in behavior of the SEO-LLTO composite and the sandwich electrolyte did not arise due to differences in electrochemical properties, we measured *κ* and *ρ*_+_ of the sandwich electrolyte. Table 2 compares these measurements with the properties of SEO and SEO-LLTO electrolytes. Both *κ* and *ρ*_+_ of the sandwich electrolyte are similar to those of the SEO and SEO-LLTO electrolytes confirming the absence of additional interfacial impedance arising in the sandwich electrolyte.

**TABLE 2:** Electrochemical characteristics of SEO-LLTO composite electrolytes at a fixed salt concentration.

Electrolyte	*κ* (mS cm^-1^)	*ρ*_+_
SEO	0.38 ± 0.05	0.05 ± 0.002
SEO-LLTO-26	0.40 ± 0.04	0.07 ± 0.02
SEO | SEO-LLTO-26 | SEO	0.42 ± 0.02	0.08 ± 0.01

## Conclusion

**FIGURE 10:**
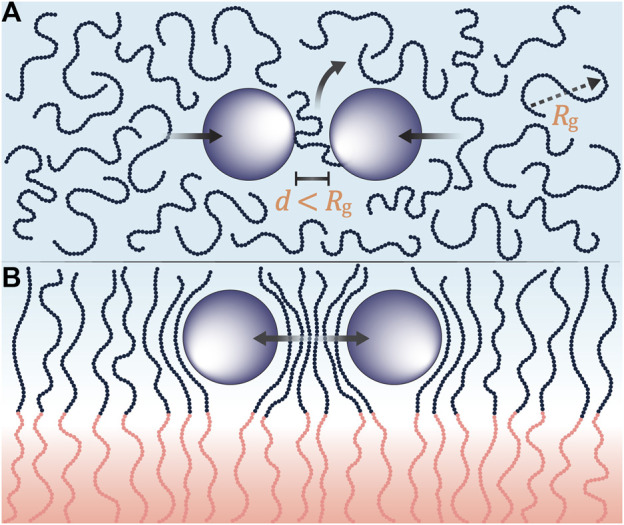
**(A)** Pictorial representation of depletion interactions where polymer chains (blue beads) with a radius of gyration, *R_g_*, are confined in between particles (purple) separated by a distance, *d*. When the *R_g_* is greater than *d*, the polymer chains are entropically driven out leading to aggregations in homopolymer composites. **(B)** Depletion interactions are suppressed when ion-conducting polymer chains are tethered to a rigid block (pink beads). As particles approach each other, the ion-conductive chains contort in entropically unfavorable configurations resulting in reduced aggregations in block copolymer composites.

We have introduced a new kind of composite electrolyte; one wherein conductive ceramic nanoparticles are introduced into the conducting lamellae of a block copolymer electrolyte. TEM images indicate a substantial reduction in nanoparticle aggregation in the block copolymer electrolyte relative to the homopolymer/nanoparticle composite (Figures 1, 2). The proposed reason for this observation is presented in Figure 10. In Figure 10A we show a homopolymer/nanoparticle composite (PEO-LLTO). In this case, the entropy of a polymer chain in the space between the particles decreases as they approach each other. This entropy decrease becomes more acute when the distance between the particle surfaces, *d*, is much smaller than the radius of gyration of the chains, *R*_*g*_. The chain gains entropy by moving away from the confining region. The net result is an entropy-based driving force that promotes particle aggregation (Gast et al., 1983). In Figure 10B we show a block copolymer/nanoparticle composite (SEO-LLTO). In this case, one end of the chains in the ion-conducting lamellae are tethered to the interface between the two microphases. Due to this, the chains must adopt distorted conformations to allow the nanoparticles to approach each other, and this reduces the entropy of the chains. The net result is an entropy-based driving force that prevents particle aggregation. While the nanoparticles were better dispersed in SEO-LLTO when compared to PEO-LLTO, many aggregates of LLTO could still be seen in SEO-LLTO. They were mainly observed at one side of the electrolyte layer. This may be due to the slowing down of drying deep in the electrolyte layer as it was prepared by solution casting.

The electrochemical properties of our SEO-LLTO composite electrolytes were similar to that of the SEO block copolymer electrolyte without nanoparticles. However, cell failure of lithium-lithium symmetric cells with the SEO-LLTO composite electrolyte occurred at very low current densities relative to SEO electrolytes without LLTO. X-ray microtomography images of failed cells show a high density of electrolyte-spanning lithium globules which seemed to envelop the LLTO aggregates, particularly those at the electrode-electrolyte interface. We also studied cell failure in lithium-lithium symmetric cells with a sandwich configuration with two SEO layers surrounding the SEO-LLTO composite. In this system, cell failure occurred at a significantly higher current density and x-ray microtomography images of failed cells show no system-spanning shorts. Our work shows the importance of eliminating aggregates, especially those at the electrode-electrolyte interface. Further work to develop processes and formulations to prevent aggregation in composite electrolytes seems warranted.

## Figures and Tables

**Table:** 

***a***	cross-sectional area of electrode (cm^2^)
***d***	particle distance (Å)
***G″***	loss modulus (Pa)
***G′***	storage modulus (Pa)
***i***	current density (mA cm^−2^)
***i***_***0***_	initial current density (mA cm^−2^)
***i***_***norm***_	normalized current density to 20 μm electrolyte (mA cm^−2^)
***i***_***ss***_	steady-state current density (mA cm^−2^)
***i***_***Ω***_	initial current corrected for ohmic losses (mA cm^−2^)
***L***	thickness of sandwiched electrolyte (μm)
***L***_***el***_	thickness of electrolyte (cm)
***M***_***EO***_	molar mass of ethylene oxide (g mol^−1^)
***m***_***LLTO***_	mass of LLTO (g)
***M***_***PEO***_	molar mass of poly(ethylene oxide) (g mol^−1^)
***M***_***PS***_	molar mass of polystyrene (g mol^−1^)
***M***_***S***_	molar mass of styrene (g mol^−1^)
***m***_***SEO***_	mass of SEO (g)
***r***	salt concentration ([Li] [EO]^−1^)
***R***_***b***_	bulk resistance (Ω)
***R***_***b,0***_	initial bulk resistance (Ω)
***R***_***g***_	radius of gyration (Å)
***R***_***i***_	interfacial resistance (Ω)
***R***_***i,0***_	initial interfacial resistance (Ω)
***T***_***g***_	glass transition temperature (°C)
***T***_***m***_	melting temperature (°C)
***w***_***LLTO***_	weight fraction of LLTO
***w***_***LLTO,PEO***_	weight fraction of LLTO with respect to PEO
Greek symbols	
$\phi_{c}$	volume fraction of the conducting phase
$\phi_{EO}$	volume fraction of ethylene oxide
$\phi_{LLTO}$	volume fraction of LLTO inside of PEO microphase
***ΔH***_***m***_	enthalpy of melting (J g^−1^)
***ΔH***_***m,PEO***_	enthalpy of melting of PEO (J g^−1^)
***ΔΦ***	applied potential (V)
***κ***	ionic conductivity (mS cm^−1^)
***ν***_***EO***_	molar volume of ethylene oxide (mol cm^−3^)
***ν***_***LiTFSI***_	molar volume of LiTFSI (mol cm^−3^)
***ν***_***LLTO***_	molar volume of lithium lanthanum titanate (mol cm^−3^)
***ν***_***S***_	molar volume of styrene (mol cm^−3^)
***ρ***_***+***_	current fraction
***χ***_***PEO***_	percent crystallinity of PEO (%)
γ	LLTO concentration ([LLTO] [EO]^−1^)

## Data Availability

The raw data supporting the conclusion of this article will be made available by the authors, without undue reservation.
