# Supplementary material for: Cycling of block copolymer composites with lithium-conducting ceramic nanoparticles
Source: Front Chem. 2023 Jun 2;11:1199677. doi: 10.3389/fchem.2023.1199677 (PMC10272992; doi:10.3389/fchem.2023.1199677)
Supplement: Supplementary file 1 [file DataSheet1.docx]

Supplementary Material

Cycling of Block Copolymer Composites with Lithium-Conducting Ceramic Nanoparticles

Vivaan Patel^1,2^, Michael A. Dato^3^, Saheli Chakraborty^2^, Xi Jiang^2^, Min Chen^5^, Matthew Moy^1^, Xiaopeng Yu^2^, Jacqueline A. Maslyn^1^, Linhua Hu^3^, Jordi Cabana^3^, Nitash Balsara^1,2,4*^

*** Correspondence:** Nitash Balsara [nbalsara@berkeley.edu](mailto:nbalsara@berkeley.edu)

# Supplementary Figures and Tables

##
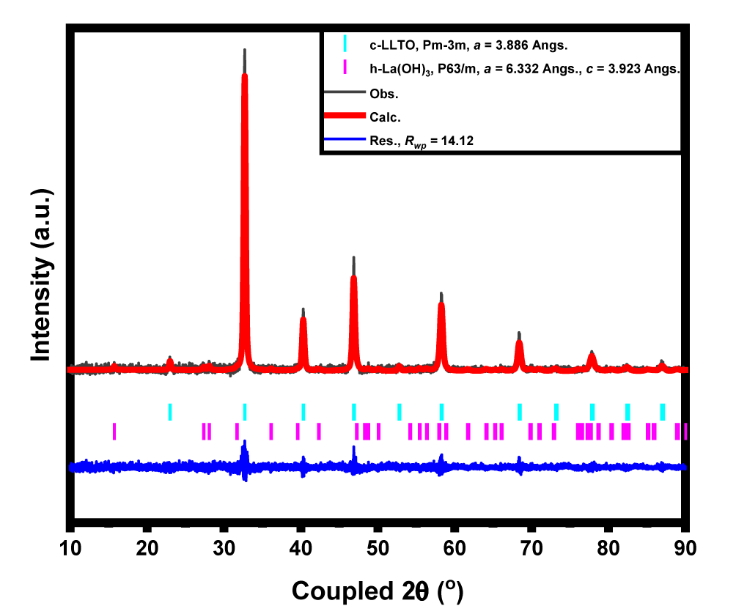
Supplementary Figures

**Supplementary Figure 1.** X-Ray Diffraction and corresponding Pawley Refinement of the cubic ceramic LLTO particles implemented into hybrid electrolytes. Powder diffraction was collected at 2theta from 10-90deg in a Bragg-Brentano geometry with a Cu-kalpha source (Kalpha1 = 1.5406 Angs., Kalpha2 = 1.5444 Angs.


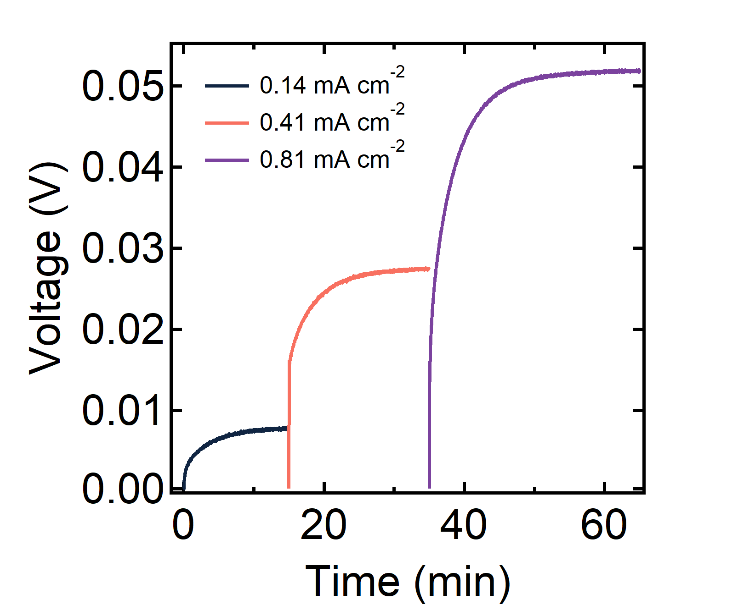


**Supplementary Figure 2.** Voltage profiles as a function of time for a layered electrolyte comprised of SEO | SEO-LLTO-26 | SEO in a lithium symmetric cell at various normalized current densities ranging from 0.14 mA cm^-2^ to 0.81 mA cm^-2^ at 90°C. The failure of any electrolyte of a given thickness, *L*, is given by the limiting current, *i*_L_, which is proportional to 1/*L*. The applied current density range for the sandwich electrolyte here corresponds to *i*/*i*_L_ comparable to the applied current densities for the SEO-LLTO-26 composite. The thickness of the sandwich electrolyte was 90 μm.
